# Identification of 4 novel human ocular coloboma genes *ANK3*, *BMPR1B*, *PDGFRA*, and *CDH4* through evolutionary conserved vertebrate gene analysis

**DOI:** 10.1016/j.gim.2021.12.014

**Published:** 2022-01-13

**Authors:** Nicholas Owen, Maria Toms, Rodrigo M. Young, Jonathan Eintracht, Hajrah Sarkar, Brian P. Brooks, Mariya Moosajee

**Affiliations:** 1 Development, Ageing and Disease, UCL Institute of Ophthalmology, London, United Kingdom; 2 Ophthalmic Genetics and Visual Function Branch, National Eye Institute, National Institutes of Health, Bethesda, MD; 3 Department of Genetics, Moorfields Eye Hospital NHS Foundation Trust, London, United Kingdom; 4 Department of Ophthalmology, Great Ormond Street Hospital for Children NHS Foundation Trust, London, United Kingdom; 5 Ocular Genomics and Therapeutics, The Francis Crick Institute, London, United Kingdom

**Keywords:** Coloboma, Congenital eye defects, Disease genetics, Genomic sequencing, Microphthalmia

## Abstract

**Purpose:**

Ocular coloboma arises from genetic or environmental perturbations that inhibit optic fissure (OF) fusion during early eye development. Despite high genetic heterogeneity, 70% to 85% of patients remain molecularly undiagnosed. In this study, we have identified new potential causative genes using cross-species comparative meta-analysis.

**Methods:**

Evolutionarily conserved differentially expressed genes were identified through in silico analysis, with in situ hybridization, gene knockdown, and rescue performed to confirm spatiotemporal gene expression and phenotype. Interrogation of the 100,000 Genomes Project for putative pathogenic variants was performed.

**Results:**

Nine conserved differentially expressed genes between zebrafish and mouse were identified. Expression of zebrafish *ank3a*, *bmpr1ba/b*, *cdh4*, and *pdgfaa* was localized to the OF, periocular mesenchyme cells, or ciliary marginal zone, regions traversed by the OF. Knockdown of *ank3*, *bmpr1b*, and *pdgfaa* revealed a coloboma and/or microphthalmia phenotype. Novel pathogenic variants in *ANK3*, *BMPR1B*, *PDGFRA*, and *CDH4* were identified in 8 unrelated coloboma families. We showed *BMPR1B* rescued the knockdown phenotype but variant messenger RNAs failed, providing evidence of pathogenicity.

**Conclusion:**

We show the utility of cross-species meta-analysis to identify several novel coloboma disease-causing genes. There is a potential to increase the diagnostic yield for new and unsolved patients while adding to our understanding of the genetic basis of OF morphogenesis.

## Introduction

Formation of the vertebrate eye involves coordinated assembly of cell types from different embryological origins and a series of tissue rearrangements. This complex process begins with the specification of eye field cells in the anterior neuroectoderm, followed by lateral migration and evagination toward the overlying surface ectoderm to form 2 optic vesicles.^1,2^ The interaction between the optic vesicles and the surface ectoderm induces the specification of lens placode, as well as initiates invagination of the optic vesicle to form a double-layered optic cup.^3^ The inner and outer layers of the optic cup give rise to the neural retina and the retinal pigment epithelium, respectively. An opening on the ventral surface of the optic cup leads to the formation of an optic fissure (OF), which enables the periocular mesenchyme (POM), composed of mesoderm and neural crest cells, to enter the developing eye and give rise to the hyaloid vasculature.^4–6^

The apposing edges of the OF begin to fuse bidirectionally from the midpoint proximally and distally, so the iris and the future optic nerve head fuse last. This process results in a continuous layered retina with no gaps. Unlike other epithelial fusion processes, which occur between the apical sides of cells, OF fusion begins with the apposition of the basal cell surface requiring breakdown of the basal lamina.^4,7,8^ An increasing number of reports show that the POM cells play a role in OF fusion, and although there is no clear mechanistic explanation, abrogation of POM specification or migration can lead to persistence of the OF.^4^

Partial or complete failure of OF fusion is known as ocular coloboma (OC) and in humans, it can lead to an inferonasal gap in the iris, ciliary body, choroid, retinal pigment epithelium, retina, and/or optic disc. This congenital eye defect has a prevalence of 7.5 in 10,000 births and accounts for 10% of childhood blindness worldwide.^5^ OC can be found in isolation, in association with other ocular malformations (complex), or as part of a syndrome with systemic features. It forms part of the clinical spectrum together with microphthalmia and anophthalmia (collectively known by the acronym MAC), with significant mixed ocular phenotypes and approximately 100 shared causative genes.^6^ Despite no notable environmental factors in pregnancy, diagnostic rates for patients remain around 10% on enriched targeted exome gene panels.^9^ Genome sequencing (GS) approaches bring the promise of increasing molecular diagnosis by a further 25% to 40%.^10^

Significant identification of genes involved in OF fusion and OC have originated from animal studies, including mice, chick, *Xenopus*, and zebrafish. We recently generated a temporal transcriptome of the developing OF in wild-type zebrafish to further our understanding of genes involved in tissue fusion events.^11^

To further identify evolutionarily conserved genes involved in OF morphogenesis and to aid the discovery of novel OC candidate genes, we undertook in silico cross-species comparative meta-analysis, mining mouse and zebrafish transcriptomes to identify conserved differentially expressed genes (DEGs).^11,12^ These genes were further studied using in situ hybridization to ascertain the spatiotemporal gene expression pattern in zebrafish, resulting in 8 potential candidates: *ank3a*, *bmpr1ba*, *bmpr1bb*, *cdh4*, *nr2f2*, *pdgfaa*, and *pdgfab*. We showed that knockdown of candidate genes can lead to failure of OF closure. Furthermore, we leveraged GS data from the 100,000 Genomes Project (launched in the United Kingdom in 2012 to improve diagnosis and clinical management of those with rare diseases or cancer)^13^ for variants in the aforementioned genes from patients with human phenotype ontology terms encompassing MAC. We found novel pathogenic variants in *ANK3*, *BMPR1B*, *CDH4*, and *PDGFRA*. In vivo zebrafish assays were performed, combining gene knockdown and human messenger RNA (mRNA) rescue to assess specificity of targeting. Variants reported in *BMPR1B* were recapitulated, and the variant mRNA failed to rescue the phenotype. We show that our approach can identify new candidate genes, which may now be included in diagnostic gene panels.

## Materials and Methods

### Zebrafish husbandry

Adult zebrafish (wild-type, AB-strain) were maintained at the University College London main campus zebrafish facility under standard husbandry conditions, and embryos were obtained by natural spawning. Wild-type and mutant embryos were raised at 28.5 °C and staged according to Kimmel et al.^14^ To minimize variations in staging, embryos were collected every 30 minutes and kept in separate clutches according to their time of fertilization. When necessary, pigmentation development was inhibited by incubating embryos in 0.2 mM phenylthiourea 24 hours post fertilization (hpf).

### Morpholino embryo microinjection and mRNA rescue

Translational blocking morpholinos (MOs) were used to knockdown endogenous zebrafish genes (Gene Tools, LLC). One to 2-cell stage embryos were coinjected with 10 nL of a mix of *GFP* mRNA (50 pg) and MOs in the amounts indicated: *ank3a* (0.33 pmol, 5′-TTTTTTCTTTGGCTTCT TCACTCAT-3′), *ank3b* (0.25 pmol, 5′-TTTTTTCTTTGG CTTCTTCACTCAT-3′), *bmpr1ba* (2 pmol, 5′-CATCGTG CGTCTGCAGAACTCCAGT-3′), *bmpr1bb* (1 pmol, 5′-TC CGTACCCGGAGCTCCATCCCTGA-3′), *cdh4* (0.1 pmol, 5′-CATGTTTAACGTTACGGGCTTGTCT-3′), *pdgfaa* (0.2 pmol, 5′-GTGGTCCAAAAGTGTCCCAGCGCAG-3′), *pdgfab* (0.2 pmol, 5′-CAATGTCAATTTCCCACCCAGCA AG-3′), and control (0.25 pmol, 5′-TGTTGAAATCAG CGTGTTCAAG-3′). Coinjection of p53 ATG-MO was used to control for p53-dependent neural toxicity. Each MO was injected into approximately 100 embryos, and the surviving ones were collected. For rescue, plasmid containing wild-type or variant *BMPR1B* was linearized by *AscI* (New England BioLabs) and heat inactivated for 20 minutes at 80 °C. Linearized complementary DNA was purified using QIAquick PCR purification kit (QIAGEN). Capped mRNAs were transcribed in vitro using MEGAscript T7 Transcription Kit (AM1334, ThermoFisher Scientific) for each template and purified using MEGAclear Transcription Clean-Up Kit (AM1908, ThermoFisher Scientific). In vitro synthesized capped mRNAs were individually coinjected with the MO for rescue experiments (75 pg).

### In situ hybridization and immunohistochemistry

Whole mount in situ hybridization was performed using digoxigenin (DIG)-labelled RNA probes according to standard protocols.^15^ Probes were synthesized using T7 or T3 RNA polymerases (Promega) according to manufacturers’ instructions and supplied with DIG labelled UTP (Roche). Probes were detected with anti-DIG-AP (1:5000, 110932 74910 Roche) or anti-DIG-POD (1:1000, 11207733910 Roche) antibodies and developed using NBT/BCIP mix (Roche) for regular microscopy or CY-3 tyramide substrate for confocal analysis. Laminin subunit alpha 1 antibody (L9393, Sigma-Aldrich) was used at 1 per 100 for whole mount immunohistochemistry using 4′,6-diamidino-2-phenylindole counterstain at 2 μM when incubating with antimouse Alexa 568 secondary antibody (A-11011, ThermoFisher Scientific) as previously described.^4^ Confocal imaging was performed on a Leica TCS SP8 confocal microscope. Image analysis measurement was performed using Fiji.^16^

### Eye measurement and statistics

Horizontal eye diameter was determined by imaging lateral views of anaesthetised larvae at 3 days after fertilization. Images were analyzed using Fiji, and for each group, the mean ± SD was reported. Data were compared using unpaired *t* tests or Mann-Whitney tests; *P* < .05 was considered to be statistically significant. For animal studies, no randomization was used and no blinding was done. The investigators were not blinded to the group allocation during experiments and/or when assessing the outcome. The variance was similar between the groups that were statistically compared.

### Patient involvement and GS

All studies conducted were approved by Moorfields Eye Hospital and the National Research Ethics Committee and were conducted in adherence to the tenets of the Declaration of Helsinki. Patients and relatives gave written informed consent for participation in this study through either the Genetic Study of Inherited Eye Disease (REC reference 12/LO/0141) or Genomics England 100,000 Genomes project (REC reference 14/EE/1112). GS was performed through the Genomics England 100,000 Genomes Project (GE100KGP) as previously described.^17^

## Results

### Comparison of conserved gene expression in vertebrate data sets

Our previous zebrafish RNA sequencing analysis established temporal and spatial expression of genes during OF closure.^11^ We identified 178 DEGs at prefusion time point (32 hpf), 78 at perifusion time point (48 hpf), and 62 at postfusion time point (56 hpf) through comparison of tissue isolated from the OF region and dorsal retina. For cross-species comparison, we used a microarray data set quantifying gene expression in mouse retinal tissue margins spanning the OF between E10.5 and E12.5,^12^ identifying 168 significantly DEGs.

**Figure 1:**
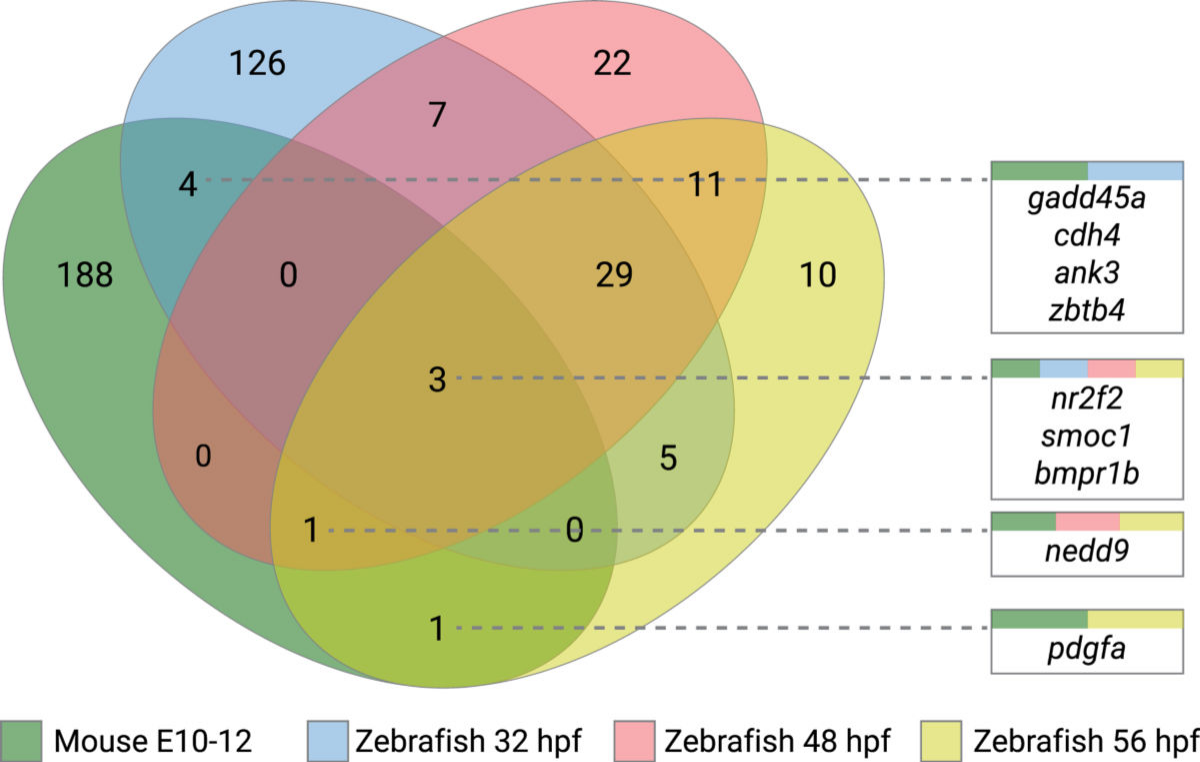
Venn diagram of the significantly regulated genes identified in mouse and zebrafish optic fissure developmental time points. The figure shows comparisons between differentially expressed gene lists of mouse and zebrafish optic fissure vs retinal tissue, with the numeric value indicating the number of genes at each intersection. Identified commonalities of genes are represented in each box, with colors representing each data set intersection. hpf, hours post fertilization.

Conservation across species within the DEG groups was confirmed and commonalities identified. Representation of common genes across species and temporal changes are shown in Figure 1 and detailed in Supplemental Table 1. We identified 9 genes that showed concordant differential expression across mouse and zebrafish OF and dorsal retina: *ank3a*, *bmpr1b*, *cdh4*, *gadd45a*, *nedd9*, *nr2f2*, *pdgfa, smoc1*, and *zbtb4*. Initially, we searched for biological significance, inspecting variant, knockout, and expression databases (Mouse Genome Informatics and The Zebrafish Information Network). We further queried clinical databases to identify genes previously associated with human variants and the MAC disease spectrum (ClinVar and Genetic Home Reference). *Smoc1* has been previously identified as a causative gene for human anophthalmia and microphthalmia,^18–20^ and zebrafish MO knockdown experiments generated a small eye phenotype but lacked a coloboma.^19^ Knockdown of *zbtb4* in zebrafish embryos showed high embryonic mortality (90%) with surviving morphants exhibiting early developmental defects, including microcephaly, which would prevent potential identification of eye defects.^21^ Therefore, both genes were excluded from further analysis.

### Phenotypic analysis of candidate gene knockdown reveals OC

**Figure 2:**
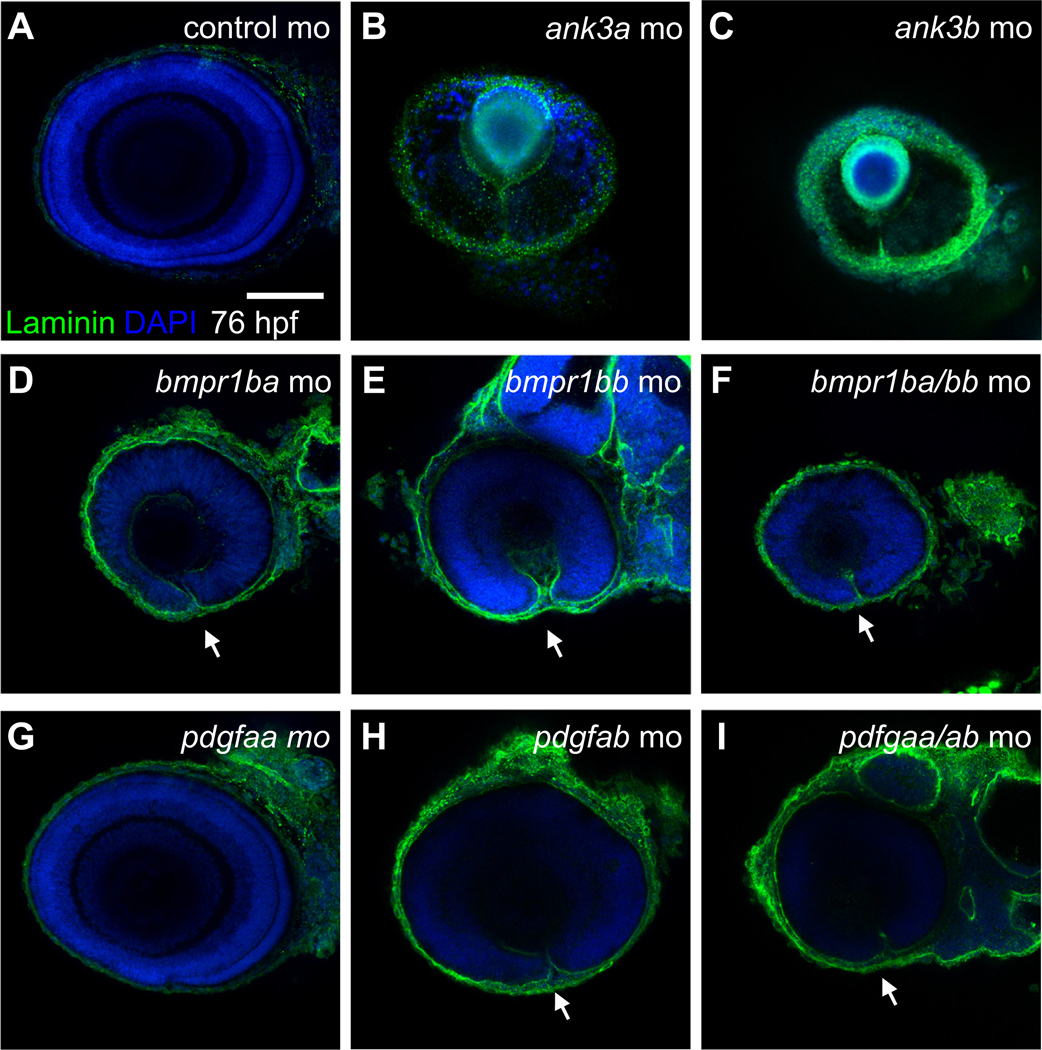
Knockdown analysis of ventral retina and optic fissure specific genes in zebrafish. Confocal imaging of lateral views (dorsal up, anterior left) of immunofluorescence staining of laminin protein in zebrafish embryos at 76 hours post fertilization in (A) control MO or MO knockdowns of (B) *ank3a*, (C) *ank3b*, (D) *bmpr1ba*, (E) *bmpr1bb*, (F) *bmpr1ba/bmpr1bb*, (G) *pdgfaa*, (H) *pdgfab*, and (I) *pdgfaa/pdgfab* mutant embryos. Scale bar = 100 μm. MO, morpholino.

To characterize loss of function (LoF), we individually knocked down the zebrafish candidate genes using translational blocking MO antisense oligonucleotides, including *ank3a*, *ank3b*, *bmpr1ba*, *bmpr1bb*, *pdgfaa*, and *cdh4*. At 56 hpf, OF fusion is complete after remodeling of the basal lamina of the apposing edges of the ventral retina with disassembly of the laminin rich extracellular matrix. Coloboma phenotypes can have a variable severity, and hence, laminin immunostaining was performed at 76 hpf to exclude the possible effect of nonspecific delay in experimental conditions. Knockdown of *ank3a* (Figure 2B; 79% penetrance, *n* = 12 embryos analyzed from 2 independent experiments) and *ank3b* (Figure 2C; 75% penetrance, *n* = 19 embryos) or both genes simultaneously (75% penetrance, *n* = 20) resulted in microphthalmia and penetrant coloboma phenotype. Laminin localization showed the position of the apposing edges of the OF. The mean eye diameter of 76 hpf *ank3a*, *ank3b*, and *ank3a/ank3b* morphants were 273 ± 27 μm, 252 ± 31 μm, and 283 ± 24 μm, respectively, compared with 308 ± 16 μm (*n* = 28) in wild-type larvae at the same timepoint (*P* < .0001 for all 3 morphant groups).

Knockdown of *bmpr1ba* (Figure 2D; 85% with coloboma penetrance, *n* = 13)*, bmpr1bb* (Figure 2E; 80% penetrance, *n* = 23), or double *bmpr1ba/bb* (Figure 2F; 1 pmol of each MO per embryo, 91% penetrance, *n* = 21) showed a penetrant coloboma covering the whole ventral retina assessed by the expression of laminin remaining along the whole proximal-distal axis of the OF. Knockdown of the *bmpr1ba*, *bmpr1bb*, and double knockdown of both genes also led to microphthalmia, with a significant reduction in eye size measured at 76 hpf compared with that of wild type. The mean eye diameter of *bmpr1ba*, *bmpr1bb*, and *bmpr1ba/bmp1bb* morphants were 230 ± 13 μm, 273 ± 28 μm, and 249 ± 18 μm, respectively (*P* < .0001 for all 3 morphant groups).

Knockdown of *pdgfaa* did not show a significant coloboma phenotype (Figure 2G; *n* = 12). However, *pdgfab* knockdown lead to mild coloboma in 50% of the embryos, with laminin expression extending through half the distalproximal axis of the ventral retina at 76 hpf (Figure 2H; *n* = 21). All double *pdgfaa/ab* morphants displayed a more severe coloboma phenotype (Figure 2I, 0.1 pmol each MO per embryo, 100%, *n* = 22). The mean eye diameter of *pdgfaa*, *pdgfab*, and *pdgfaa/pdgfab* morphants were 302 ± 12 μm, 284 ± 19 μm, and 267 ± 24 μm, respectively (*P* = 0.04, *P* < .0001, and *P* < .0001 respectively) indicating microphthalmia.

*cdh4* knockdown with 0.2 pmol MO per embryo led to pleiotropic developmental defects with embryonic maldevelopment and necrosis as previously described.^22^ To circumvent this, embryos were injected with 0.1 pmol MO, which revealed a colobomatous defect at 76 hpf (65% penetrance, *n* = 22, Figure S3). *cdh4* morphants displayed microphthalmia with a mean eye diameter of 227 ± 19 μm (*P* < .0001 compared with controls).

### In silico identification of candidate gene variants in patients with ocular maldevelopment

This study was undertaken as part of the Genomics England Clinical Interpretation Partnership to analyze genomic data from the 100,000 Genomes Project.^13^ We screened GS data to identify putative novel genes that may cause MAC phenotypes. Using UK 100,000 Genomes Project Data Release 8.0 (November 28, 2019) we identified 325 unsolved patients suitable for screening and variant prioritization. Table 1 presents a summary of our findings. Six families were identified with a diagnosis of OC and/or microphthalmia and likely pathogenic variants in *ANK3, BMPR1B, CDH4*, and *PDGFRA*. Variants were predicted to be disease causing/damaging with low or no allele frequency in Genome Aggregation Database or the Human Genetic Variation Database, classified on the basis of the American College of Medical Genetics and Genomics/Association of Molecular Pathology (ACMG/AMP) criteria.^23^ The probability of a gene being LoF intolerant (pLI) scores were reported as *ANK3* (pLI 1), *BMPR1B* (pLI 1), *CDH4* (pLI 0.73), and *PDGFRA* (pLI 1). No putative pathogenic variants were detected in *GADD45A*, *NEDD9*, *NR2F2*, and *PDGFA* genes. Deleterious variants, including structurals and copy number variations, were not detected in genes previously known to cause MAC in these subjects (PanelApp – Genomics England, Anophthalmia or microphthalmia [Version 1.42], Ocular coloboma [Version 1.44] gene panels).

**Figure 3:**
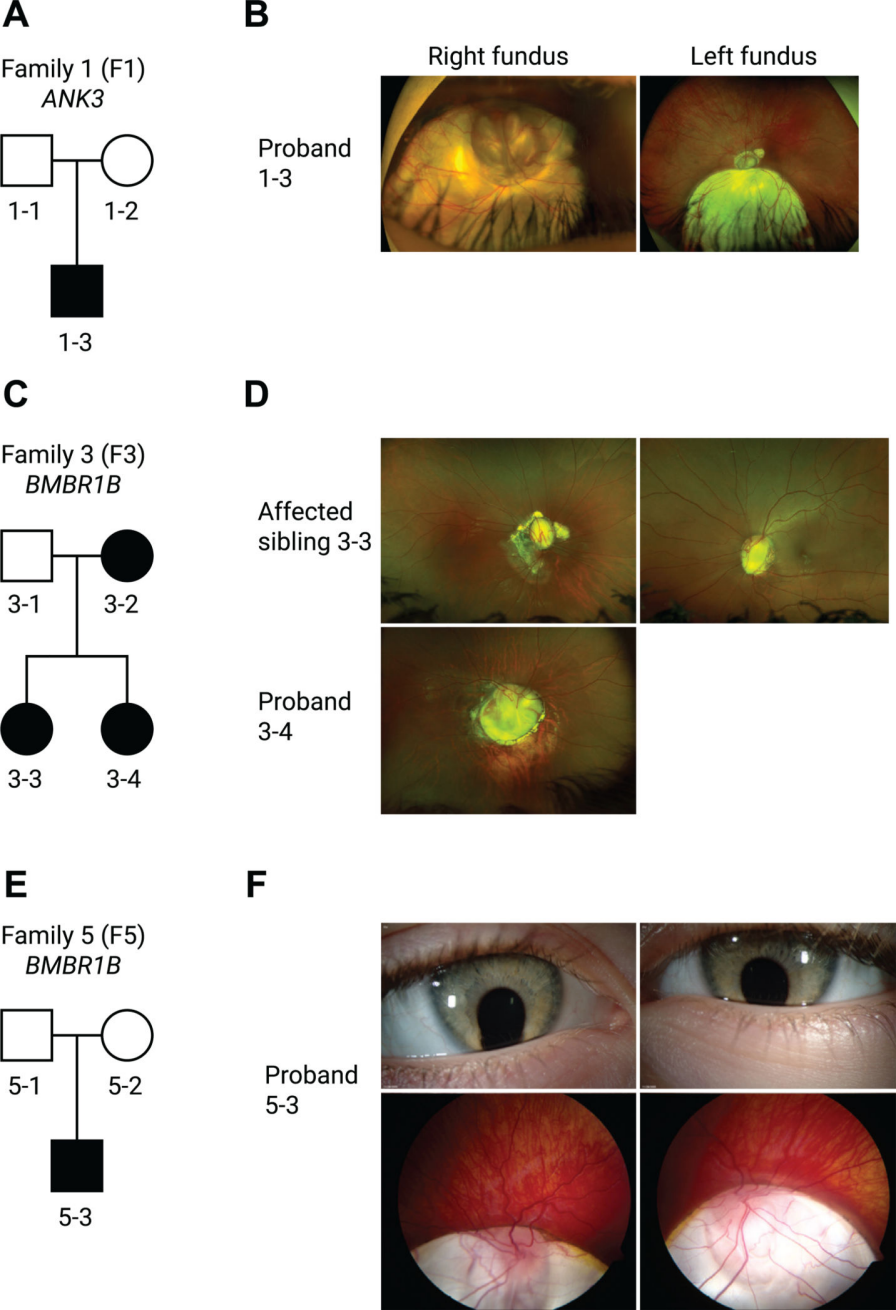
Pedigrees and clinical imaging of 3 unrelated coloboma families. A. F1 is a White British nonconsanguineous pedigree with 1 affected male patient (proband 1–3) harboring a de novo sporadic heterozygous missense variant in *ANK3* NM_020987.5:c.11650A>T, p.(Thr3884Ser). B. Corresponding widefield color fundus photographs of the right and left eye of the proband aged 17 years showing bilateral inferior chorioretinal coloboma with extensive optic disc involvement (right eye is more severely affected than the left eye as indicated also by the residual best corrected visual acuity). C. F3 is an Indian nonconsanguineous pedigree with autosomal dominant inheritance; the mother and the 2 daughters were affected with a heterozygous missense variant in *BMPR1B* NM_001203.2:c.272G>T, p.(Arg91Ile). D. Widefield color fundus photograph of the right eye of the proband (3–4) aged 13 showed a large optic disc coloboma extending inferiorly with a region of associated retinal pigment epithelium atrophy below this. The left eye image was not available. Widefield color fundus photographs of the right and left eye of the proband (3–3) aged 18 showing bilateral optic disc coloboma sparing the macula, with irregular asymmetrical peripapillary atrophy in the right eye. E. F5 is a White Northern European nonconsanguineous pedigree with 1 affected male patient (proband 5–3) with a heterozygous missense variant in *BMPR1B* NM_001203.2:c.671 G>A, p.(Arg224His). Parental samples for segregation were not available. F. Anterior segment color photographs of the right and left eye of the proband aged 4 showing bilateral inferior iris coloboma and corresponding widefield color fundus photographs showing bilateral inferior chorioretinal coloboma with extensive optic disc and macular involvement. F1, family 1; F3, family 3; F5, family 5.

We had access to families recruited into the 100,000 Genome Project through Moorfields Eye Hospital and Great Ormond Street Hospital for Children. Supplemental Table 2 summarizes the clinical details of 8 unrelated families. Family 1 (nonconsanguineous, White) had a proband with no previous family history, and segregation of parents was negative, indicative of a de novo sporadic heterozygous missense variant in *ANK3* NM_020987.5:c.11650A>T, p.(Thr3884Ser). Consistent with the zebrafish morphants, the patient (P1) displayed bilateral chorioretinal coloboma involving the optic discs (Figure 3A and B). The variant was classified as pathogenic on the basis of ACMG/AMP criteria with evidence levels pathogenic strong (PS)-2, pathogenic moderate (PM)-2, pathogenic supporting (PP)-3, and PP4. Family 2 (nonconsanguineous, Black) proband was a male, presenting with bilateral optic nerve hypoplasia and left optic disc coloboma, and had a heterozygous variant in *ANK3* NM_020987.5:c.3658A>G, p.(Ile1220Val) identified and classified as pathogenic on the basis of ACMG/AMP criteria (PS2, PM2, PP2/3/4); the unaffected mother did not carry the variant. Family 3 (nonconsanguineous, Indian) showed an autosomal dominant inheritance with 2 affected siblings and the mother with the confirmed heterozygous missense *BMPR1B* variant NM_001203.2:c.272G>T, p.(Arg91Ile), and each recorded to have bilateral optic disc coloboma (Figure 3C and D). The variant was classified as pathogenic on the basis of ACMG/AMP criteria with evidence levels PS2, PM2, PP3, and PP4. Family 4 (non-consanguineous, White) consisted of a single proband and no family history, and segregation of parents was negative; a de novo sporadic heterozygous missense variant was found in *BMPR1B* NM_001203.2:c.1127G>A, p.(Arg376Glu). This patient had a unilateral right microphthalmia, right dense cataract with no view of the fundus, but at age 5 weeks he underwent an examination under anesthesia, which reported persistent hyperplastic primary vitreous. The variant was classified as pathogenic on the basis of ACMG/AMP criteria (PS2, PM1, PM2, PP3, and PP4). Collaboration with the National Eye Institute aided the identification of a 4-year-old White male with bilateral iris and chorioretinal coloboma harboring a variant in *BMPR1B* exon 7; c.671G>A, p.(Arg224His) (family 5, Figure 3E and F). The variant was classified as pathogenic on the basis of ACMG/AMP criteria (PS2, PM1, PM2, PP4). In addition, in an unrelated 10-month-old White male (family 6) with right iris and bilateral chorioretinal coloboma with a c.671G>T, p.(Arg224Leu) variant in exon 7 of *BMPR1B*, this variant was classified as likely pathogenic on the basis of ACMG/AMP criteria (PS2, PM2, PP2, PP3, and PP4). Two more variants were identified in families within Genomics England 100,000 Genomes Project external to the Moorfields Eye Hospital clinic; family 7 proband presented with bilateral chorioretinal coloboma and microphthalmia as well as global developmental delay with autistic behavior and harbored a heterozygous variant in *PDGFRA* NM_006206.6:c.1295C>T, p.(Thr432Met) classified as likely pathogenic on the basis of ACMG/AMP criteria (PS2, PM2, PP2, PP3, and PP4), and the unaffected mother was homozygous for the wild-type reference sequence. Family 8 proband presented with an iris coloboma, intellectual disability, and microcephaly, in which we identified a heterozygous variant *CDH4* NM_001794.5:c.1291C>T, p.(Arg431Cys), which was classified as likely pathogenic (PS2, PM2, PP2, PP3 and PP4), and the unaffected mother was homozygous for the reference sequence. Supplemental Table 3 outlines the classification criteria.

### Human *BMPR1B* rescues morphant phenotype in zebrafish

**Figure 4:**
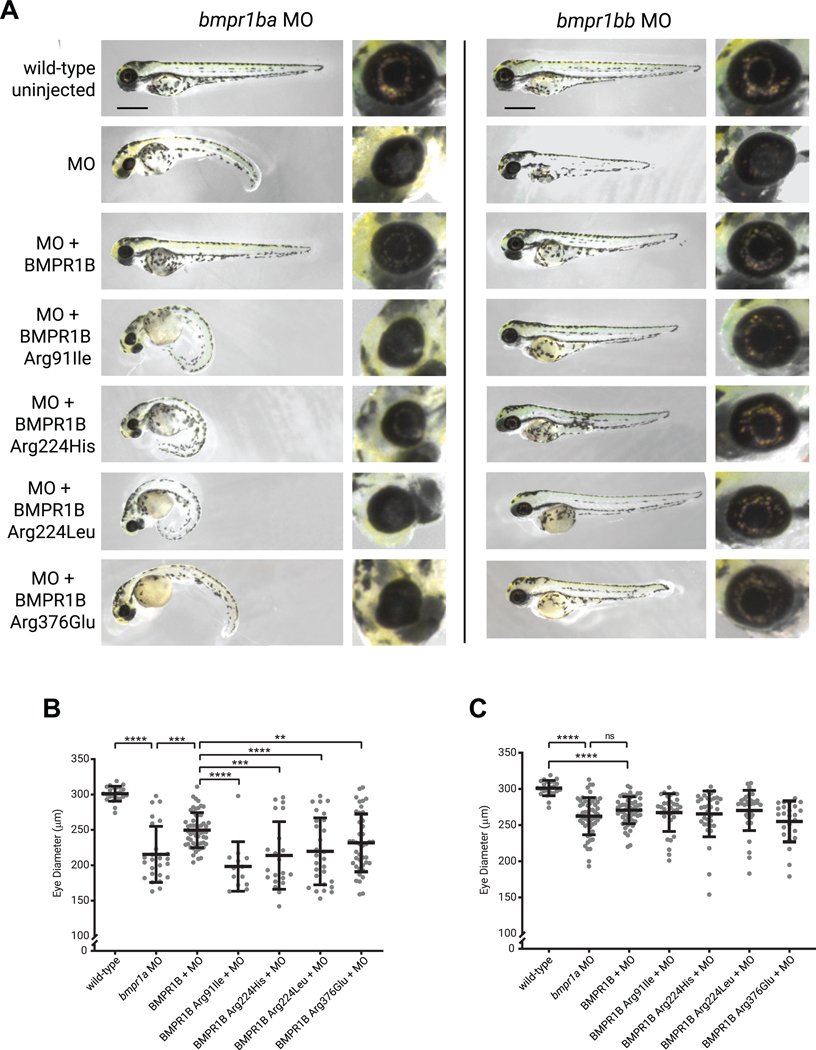
MO knockdown and rescue experiments of *bmpr1ba/b* in zebrafish. Injection of the MOs alone showed reduction in eye size and the presence of a coloboma. A. Coinjection with in vitro synthesized human *BMPR1B* messenger RNA (mRNA) rescues the phenotypic effects of the MO alone. Human variants identified within the Genomics England cohort were expressed and mRNAs coinjected with each MO. Scale bar 500 μm. Eye diameter was reported for (B) *bmpr1ba* and (C) *bmpr1bb* compared with wild-type uninjected; MO; MO + human wild-type *BMPR1B*; and variants Arg91Ile, Arg224His, Arg224Leu, and Arg376Glu. (*n* for *bmpr1ba* experiments = 23, 25, 55, 13, 21, 28, 42 and *n* for *bmpr1bb* experiments = 23, 56, 55, 32, 34, 33, 24). Unpaired *t* tests were used to compare data. **P < .01, ***P < .001, ****P < .0001, ns *P* > .05. MO, morpholino; ns, nonsignificant.

To validate the consequences of the *bmpr1b* MO effect, we employed a rescue approach with coinjection of human *BMPR1B* mRNA. The severe phenotype of *bmpr1ba* MO was rescued through concomitant microinjection of human wild-type *BMPR1B* mRNA, wherein the exogenous expression of wild-type *BMPR1B* was able to restore eye diameter from mean 215 ± 4 μm to 250 ± 2 μm (Figure 4A). Uninjected wild-type controls had an eye diameter of 301 ± 11 μm. No rescue of the phenotype was observed in embryos coinjected with mRNA carrying the patient-specific variants identified (c.272G>T, p.[Arg91Ile], 198 ± 3 μm; c.671G>A, p.[Arg224His], 214 ± 5 μm; c.671G>T, p.[Arg224Leu], 220 ± 5 μm; and c.1127G>A, p.[Arg376Glu], 232 ± 4 μm) (Figure 4B), confirming the LoF effect of the variants on BMPR1B function. The morpholino phenotype was less severe for *bmpr1bb*, and although remaining significant, rescue with human *BMPR1B* had a less pronounced effect (Figure 4C).

## Discussion

In this investigation, we have shown that the study of genes with evolutionarily conserved expression identified by comparing model system expression data sets is a powerful approach to discover new variants involved in human diseases. It has led to the discovery of 4 novel OC genes *ANK3*, *BMPR1B*, *CDH4*, and *PDGFRA*, with pathogenic variants of diagnostic value not previously reported in this patient cohort. There is significant genetic and phenotypic heterogeneity in patients with MAC. It is rarely an isolated pure disorder but more commonly found as a mixed presentation with between 33% and 95% of cases displaying additional nonocular systemic malformations.^6^ Genes coordinating optic vesicle and fissure morphogenesis are time- and dose-critical. Genetic perturbations can have a global effect on ocular development with most variants associated with nonsyndromic MAC arising as a de novo sporadic .^24^ With these dominant heterozygous variants, variable expressivity and penetrance can be seen, and this may be linked to genetic modifiers and/or stochastic environmental factors, which influence the levels of gene expression. Unilateral ocular phenotypes were seen in patients 3–2, 4–1, and 8–1. Such phenotypic variation is not an uncommon finding in individuals with molecularly-confirmed isolated and syndromic MAC. Several hypotheses have been put forward to explain this laterality despite the presence of germline variants, including an intrinsic sensitivity of the developing eye to stressors, suggesting that a potential hypomorphic allele may be more penetrant in ocular tissues than systemically. A study identified 3 human patients with unilateral microphthalmia caused by germline *TMX3* variants and postulated that the asymmetry was due to a delay in eye formation and that reduced gene dosage could be compensated for by different genes.^25,26^

The BMP signaling pathway has been previously related to eye malformations.^6,24^ BMP signaling is known to work at 2 crucial stages of eye formation, at eye field specification during gastrulation^1^ and the process of optic cup morphogenesis.^2^ For example, *BMP4* LoF results in a MAC phenotype, whereas *BMP7* LoF specifically results in coloboma.^27,28^ A recent study has reported a mouse model harboring an N-ethyl-N-nitrosourea (ENU) derived *Bmpr1b* variant showing optic nerve coloboma, with gliosis of the ventral retina.^29^ Our experiments in zebrafish show that inhibition of *bmpr1ba/bb* leads to embryos with coloboma and microphthalmia. This is in line with previous reports that have shown that impairment of the BMP pathway can lead to a persistent OF and morphological defects of the optic cup because it may be required for the migration of optic stalk cells into the anterior neural retina.^30,31^ Importantly, our study reveals that human BMPR1B performs a similar function to zebrafish protein because the human wild-type BMPR1B can compensate for the knockdown in zebrafish morphants. However, variant proteins (Arg91Ile, Arg224His, Arg224Leu, and Arg376Glu) cannot rescue the morphant phenotype, suggesting these variants interfere with *BMPR1B* function.

The ankyrins are a family of proteins whose key role is linking membrane-spanning proteins to the underlying actin cytoskeleton. Ankyrins have been shown to modulate cellular organization; AnkG (encoded by the *Ank3* gene) is specifically required for epithelial cell polarity, proliferation, and cell survival.^32^ A key protein partner of AnkG is SMAD2, one of the main transcription factors in the canonical TGF-β signaling pathway, which is involved in direct signaling from TGF-β receptors in BMP pathways.^31^ Modulation of BMP signaling by variants in *YAP1* via interaction with SMAD7, a major contributor to the BMP pathway, has been shown to result in OF closure defects, including coloboma.^33^ It is therefore plausible that variants in *ANK3* result in altered BMP signaling through a similar manner, altering SMAD7 regulation through the interaction with SMAD2 and leading to the OC observed in this study. Further investigation of SMAD2/7 expression patterns in *ank3a/b* morphants would be worth evaluation.

PDGF signaling has been related to neural crest migration and vasculature formation in early development.^34,35^ The POM cells are neural crest derivatives that give rise to the retinal vasculature that enters through the OF and later contribute to anterior segment development.^36,37^ Depletion of POM cells from zebrafish optic vesicles have resulted in failure of OF closure.^4^ PDGF activates a cellular response through cell surface receptors PDGFR(A/B), binding to cofactors and activating signal transduction. PDGFRs are involved in several signaling pathways: RAS/MAPK, STAT3, and VEGF. Increased apoptosis on the pathway of migrating neural crest cells was observed in *Pdgfra* knockout mice, and reduced capacity for migration and neuronal differentiation was reported in the *Pdgfrb* knockout mice.^38^ The inhibition of PDGFA leads to OC by potentially disrupting the migration of cranial neural crest cells into the eye.

Cadherin adhesion molecules are required for eye development, and variants in *CDH23* and *PCDH15* can lead to inherited eye disorders such as Usher syndrome.^39^ Developmental eye defects such as microphthalmia, cataracts, and microspherophakia and lack of retinal lamination have been previously shown in MO knockdown of *cdh4* in zebrafish.^22^ Reduced dose knockdown of *cdh4* in this study resulted in overall normal embryo development with no evident necrosis but a resultant coloboma phenotype. Laminin expression was downregulated suggesting a regulatory role in this pathway. Laminin variants (subunits beta-1 and gamma-1) have been found to display colobomatous defects in zebrafish,^40^ but no corresponding human variants have been previously described with this phenotype. Of note, the proband identified in family 8 presented with intellectual disability and postnatal microcephaly; similarly, the *cdh4* morphant phenotype at standard dose presented with pleiotropic developmental defects. Knockdown of *cdh4* in zebrafish results in evidence of brain disorganization.^22^

Overall, by studying genes that show an evolutionarily conserved expression in the eye, we have generated a pipeline that has allowed the identification of new candidate disease genes. Most patients with MAC harbor de novo sporadic heterozygous variants that lead to dominant inheritance, which has implications for future generations. Improving diagnostic capabilities will allow for informed genetic counselling and family planning, such as preimplantation diagnosis or noninvasive prenatal diagnosis. Using transcriptome data sets may allow us to compare genes involved in other tissue fusion events such as the neural tube, providing further universal gene candidates. Future investigation of functional pathways may contribute to the development of preventative therapeutic interventions, similar to folic acid supplementation, which may limit the severity of tissue closure defects.

## Supplementary Material

supplementary material

## Figures and Tables

**Table 1:** Summary of *in silico* analysis of single nucleotide variants (SNVs) identified

Fs	HPO Terms	Gene	cDNA / GRCh38	Protein	Exon	SIFT	PolyPhen-2	MutationTaster	CADD	gnomAD

F1	Visual impairment, retinal coloboma, iris coloboma, chorioretinal coloboma	*ANK3*	NM_020987.5 c.11650 A>T 10:60069231:T:A	p.(Thr3884Ser)	37	Damaging	Probably damaging	Disease causing	15.12	<0.002%
F2	Visual impairment, coloboma, global developmental delay, micropenis	*ANK3*	NM_020987.5 c.3658 A>G 10:60086767:T:C	p.(Ile1220Val)	30	Damaging	Damaging	Disease causing	25.8	<0.002%
F3	Visual impairment, optic nerve coloboma	*BMPR1B*	NM_001203.2 c.272 G>T 4:95115710:G:T	p.(Arg91Ile)	6	Damaging	Probably damaging	Disease causing	27.9	<0.007%
F4	Visual impairment, microphthalmia	*BMPR1B*	NM_001203.2 c.1127 G>A 4:95148798:G:A	p.(Arg376His)	11	Deleterious	Probably damaging	Disease causing	32.0	NF
F5	Bilateral iris coloboma and chorioretinal coloboma	*BMPR1B*	NM_001203.2 c.671 G>A 4:95129947:G:A	p.(Arg224His)	9	Deleterious	Benign	Disease causing	25.8	<0.096%
F6	Iris coloboma, chorioretinal coloboma	*BMPR1B*	NM_001203.2 c.671 G>T 4:95129947:G:T	p.(Arg224Leu)	9	Deleterious	Probably damaging	Disease causing	31	NF
F7	Visual impairment, bilateral microphthalmos, bilateral retinal coloboma	*PDGFRA*	NM_006206.6 c.1295 C>T 4:54272451:C:T	p.(Thr432Met)	9	Damaging	Probably damaging	Disease causing	13.27	<0.002%
F8	Iris coloboma	*CDH4*	NM_001794.5 c.1291 C>T 20:61910524:C:T	p.(Arg431Cys)	9	Damaging	Benign	Disease causing	24.5	NF

Screening of probands enrolled in the 100,000 Genomes Project presenting with HPO terms, including microphthalmia, anophthalmia, or coloboma, for putative damaging variants resulted in identifying 8 families. Position of the SNV in the cDNA and subsequent amino acid change along with the genomic location for the human reference genome GRCh38 are presented in the table. All variants identified were heterozygous. Outcomes of predictive algorithms SIFT, PolyPhen-2, and MutationTaster are reported, with respective scaled CADD scores. Allele frequency was identified in gnomAD (accessed January 2020). Two more Fs (F5 and F6) were detected through international collaboration.

*CADD*, Combined Annotation Dependent Depletion; *cDNA*, complementary DNA; *F*, family;*gnomAD*, Genome Aggregation Database; *HPO*, Human Phenotype Ontology; *PolyPhen*, Polymorphism Phenotyping; *NF*, not found; *SIFT*, Sorting Intolerant from Tolerant; *SNV*, single nucleotide variant.

## Data Availability

The expression data used in this manuscript are at Gene Expression Omnibus under the accession numbers GSE159822 and GSE13103 and are currently open access.
