# Supplementary material for: Identification of 4 novel human ocular coloboma genes ANK3, BMPR1B, PDGFRA, and CDH4 through evolutionary conserved vertebrate gene analysis
Source: Genet Med. Author manuscript; Available in PMC 2024 Oct 25. (PMC11505079; doi:10.1016/j.gim.2021.12.014)
Supplement: supplementary material [file NIHMS2015981-supplement-supplementary_material.pdf]

Identification of four novel human ocular coloboma genes *ANK3*, *BMPR1B*, *PDGFRA* and *CDH4* through evolutionary conserved vertebrate gene analysis

Supplementary Information

1. **Supplementary Methods**
2. **Supplementary Results**
3. **Supplementary Figures**
4. **Supplementary Tables**
5. **Supplementary References**

1. Supplementary Methods

In silico analysis

The *in silico* analysis of the mouse and zebrafish datasets was performed using R. Differentially expressed genes were identified and annotated using biomaRt homolog attributes (mmusculus/drerio_homolog_gene_name/chromosome/gene_id) for orthologous genes in each species. In the case where no orthologue was identified, multiple sequence alignment was carried out using Clustal Omega to classify candidate orthologous sequences. Commonalities of approved gene names were used to indicate conserved genes of interest.

Site-directed mutagenesis

cDNA encoding full length human *BRMP1B* cloned into the pReceiver-B02 vector (Genecopoeia) was used to synthesise capped mRNA for the zebrafish rescue experiments. Site-directed mutagenesis was performed using QuickChange ® II XL Site-Directed Mutagenesis kit (Aligent Technologies), introducing sequence variants into the WT cDNA, confirmed by bidirectional Sanger sequencing. The following mutagenesis primers were used to generate *BMPR1B* c.272G>T (forward 5'-CAC TCC CAT TCC TCA TCA AAG AAT ATC AAT TGA ATG CTG CAC-3, reverse 5'-GTG CAG CAT TCA ATT GAT ATT CTT TGA TGA GGA ATG GGA GTG-3'), c.1127G>A (forward 5'-TCG AGT TGG CAC CAA

ACA CTA TAT GCC TCC AGA AG-3', reverse 5'-CTT CTG GAG GCA TAT AGT GTT TGG TGC CAA CTC GA-3'), c.671G>A (forward 5'-GGA TGG GAA AGT GGC ATG GCG AAA AGG TAG C-3', reverse 5'-GCT ACC TTT TCG CCA TGC CAC TTT CCC ATC C-3'), and c.671G>T (forward 5'-GGA TGG GAA AGT GGC TTG GCG AAA AGG TAG C-3', reverse 5'-GCT ACC TTT TCG CCA AGC CAC TTT CCC ATC C-3').

Patient involvement and whole genome sequencing

Genomic DNA was processed using TruSeq DNA PCR-Free Sample Preparation kit and sequenced via the high-throughput HiSeq X Ten sequencing platform (Illumina Inc.) producing a minimum coverage of 15X for >97% of the callable autosomal genome. Reads were aligned to the human genome (GRCh37 or GRCh38) using Isaac (Illumina Inc.) and single-nucleotide variants (SNVs) and indels (insertions or deletions) were called using Starling (v2.4.7, Illumina Inc.) Copy number variants (CNVs) were reported using Canvas, and Manta called structural variants (SVs). To identify unsolved probands presenting with a MAC-related phenotype we interrogated the Research Environment Labkey sample database for human phenotype ontology (HPO) terms including microphthalmia, anophthalmia, or coloboma. Coordinates of the coding regions of genes of interest were identified and variant call files (vcf; single nucleotide variants/small indels, and structural variants) were positionally filtered. Extracted variants were annotated using Ensembl Variant Effect Predictor (VEP) and prioritised for characteristics such as allele frequency (<0.02%), consequence type, deleteriousness (as predicted via SIFT, Polyphen-2 and MutationTaster), inheritance pattern where familial samples were available, location within the gene of interest, Combined Annotation Dependent Depletion (CADD[1] scores, population frequency via gnomAD, using stepwise filtering. The presence of variants was manually inspected using Integrative Genomics Viewer (IGV).

2. Supplementary Results

Whole mount in situ hybridization gene expression analysis in the zebrafish eye.

We performed *in situ* hybridisation of *ank3a, bmpr1ba, bmpr1bb, cdh4*, *gadd45a*, *nedd9*, *nr2f2, pdgfaa and pdgfab* genes to validate their spatial expression pattern in the eye and POM to generate a hypothesis on their putative function in the OF fusion process. Wild-type zebrafish embryos (n=3) were analysed at pre-fusion 32 hpf (Fig. S1A,D,G,J,M), peri-fusion 48 hpf (Fig. S1B,E,H,K,N) and post-fusion 56 hpf (Fig. S1C,F,I,L,O) timepoints for each gene.

The expression of the zebrafish homologue of *nr2f2* was detected at low levels throughout the eye with slightly increased expression in the dorsal retina at all stages (Fig. S2A-C). *gadd45a* did not show any retinal-specific expression during OF morphogenesis (Fig. S2D-F). *nedd9* was mildly expressed in the dorsal retina, excluded from the OF region at 32 hpf, with no expression at later timepoints (Fig. S2G-I). Knockout of the mouse *Nr2f1/2* genes in combination has previously demonstrated a dramatic coloboma and microphthalmia phenotype [2]. Therefore, these genes were not investigated further in the zebrafish but were interrogated in the human dataset, in case of inter-species effects.

ank3a showed localised expression throughout the lens at 32 hpf (Fig. S1A) and then diffuse expression in the retinal ganglion cell layer at 48 hpf and 56 hpf (Fig. S1B,C). The zebrafish genome includes a duplicate of the human and mouse *Bmpr1b* homologue, *bmpr1ba* and *bmpr1bb*. Both genes were shown to display localised expression at the edges of the OF in the ventral retina (Fig. S1D-I, arrows). Even at 56 hpf, expression persisted in the region of

the fused retina. *cdh4* showed a weak diffuse expression pattern throughout the eye at 32 hpf (Fig. S1J), but at 48 and 56 hpf it became localised circumferentially to the retinal ganglion cell layer (Fig. S1K,L, arrows). *pdgfaa* expression was not detected in the optic cup at any timepoint, however, it was visualised in the POM at 32 hpf as previously described [3] (Fig. S1M-O). Given that the POM has been shown to have a role in OF closure, the function of this gene was further analysed [4,5].

3. Supplementary Figure Legends

Fig S1. Whole mount *in situ* hybridisation of ventral retina candidate genes in zebrafish. Confocal imaging of lateral views (dorsal up, anterior left) of zebrafish wild-type embryos at 32, 48 and 56 hpf, detecting the fluorescent *in situ* hybridisation signal of *ank3a* (A-C), *bmpr1ba* (D-F), *bmpr1bb* (G-I), *cdh4* (J-L), *pdgfaa* (M-O). Arrows indicate the position of the expression of genes in the area of the optic fissure (E-I), ciliary marginal zone (K, L) or periocular mesenchyme cells (M). Scale bar = 100 μm.

Fig S2. Whole mount *in situ* analysis of ventral retina and optic fissure specific genes in zebrafish. Confocal images of zebrafish wild-type embryos at 32, 48 and 56 hpf, detecting the fluorescent *in situ* hybridisation signal of *nr2f2* (A-C), *gadd45a* (D-F), *nedd9* (G-I). Representative lateral views (dorsal up, anterior left). Scale bar=100 μm.

Fig S3. Knockdown analysis of *cdh4* in zebrafish. Bright field images of representative zebrafish wild-type embryo (A) at 72 hpf and treated with *cdh4* morpholino (B). Scale bar=300 μm.

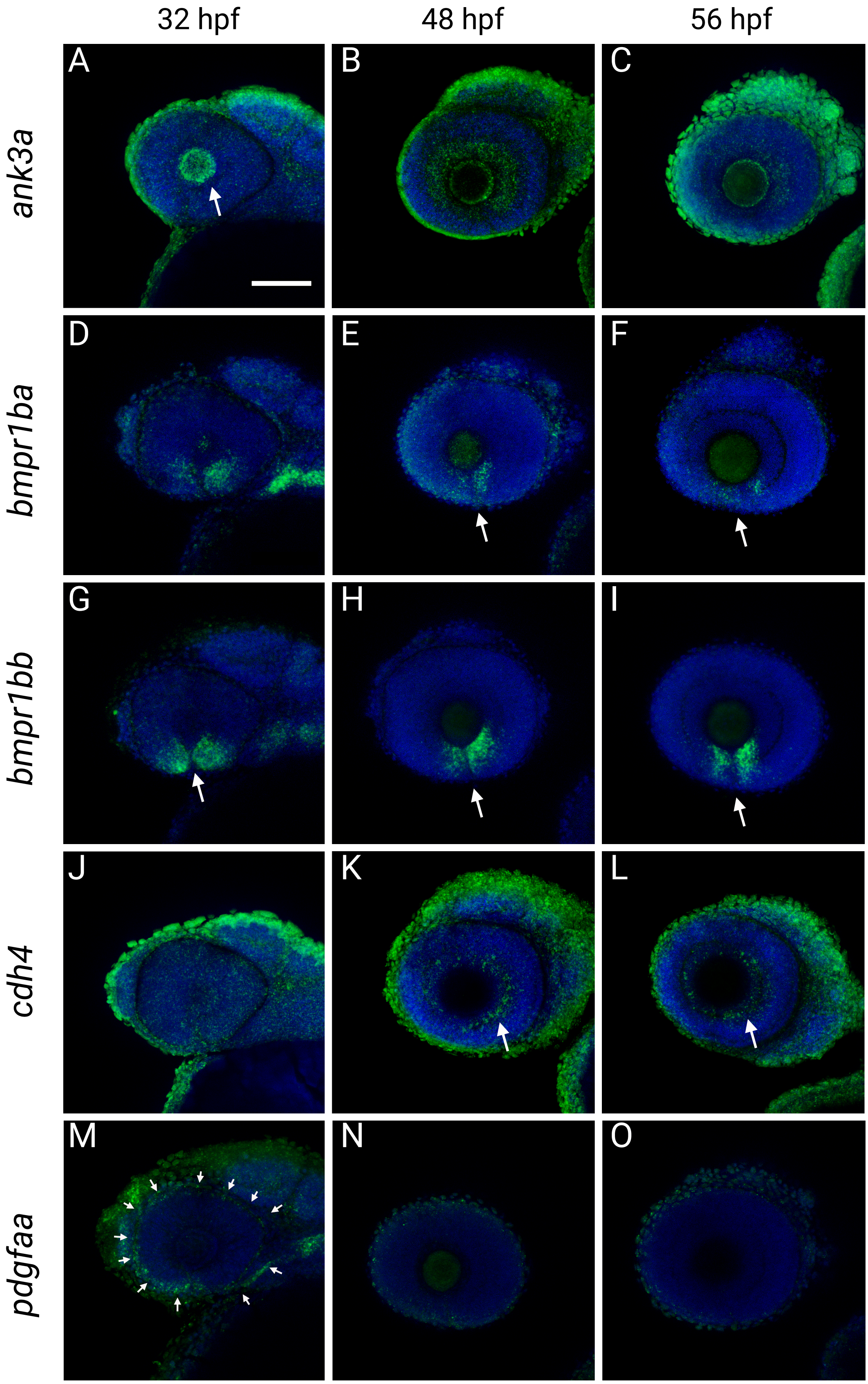

32 hpf
48 hpf
56 hpf
A
B
C
D
E
F
G
H
I
J
K
L
M
N
O
ank3a
bmpr1ba
bmpr1bb
cdh4
pdgfaa

Figure S1

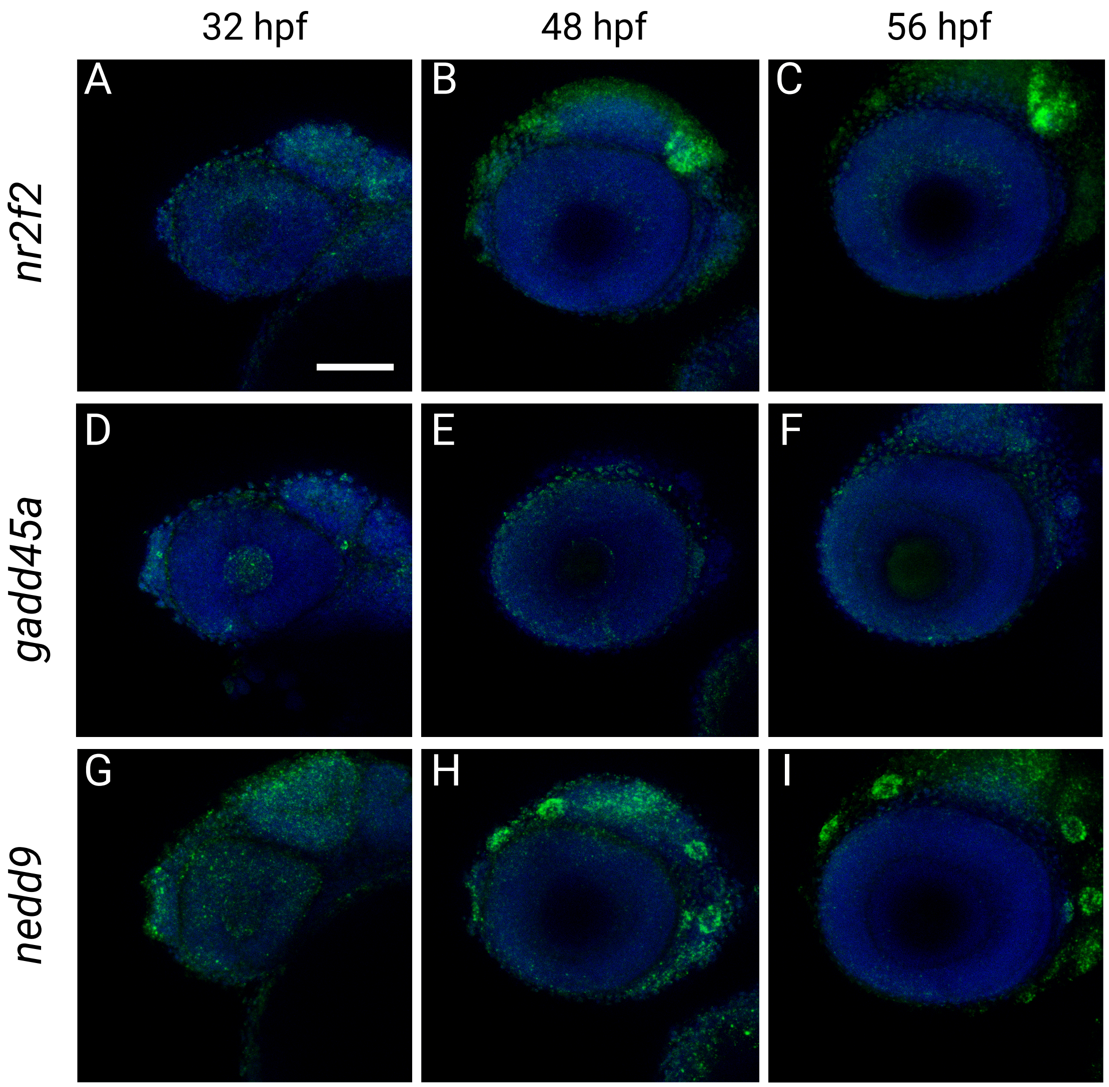

Figure S2

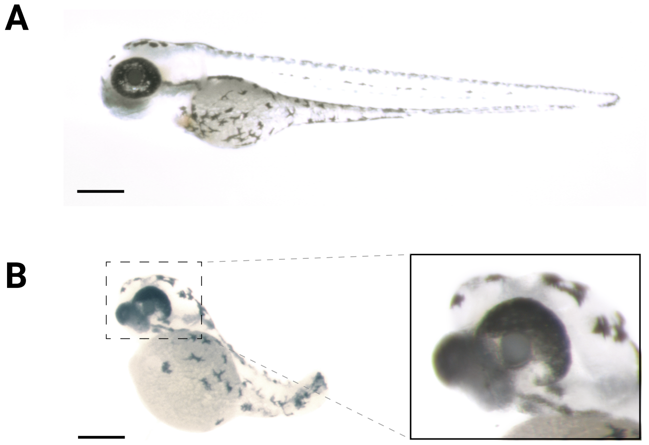

Figure S3

4. Supplementary Tables

Gene	Ensembl ID	ZF LFC OF vs DR	FDR	hpf
smoc1	ENSDARG00000088255	9.54	2.14E-13	32
nr2f2	ENSDARG00000040926	-2.57	1.24E-15	
bmpr1ba	ENSDARG00000104100	2.28	4.73E-06	
gadd45ab	ENSDARG00000104571	2.06	1.06E-05	
cdh4	ENSDARG00000015002	1.93	2.88E-04	
zbtb4	ENSDARG00000105255	1.64	1.59E-03	
ank3a	ENSDARG00000061736	3.06	2.52E-03	
smoc1	ENSDARG00000088255	5.14	3.39E-09	48
nr2f2	ENSDARG00000040926	-1.63	1.06E-05	
nedd9	ENSDARG00000089878	-1.28	2.98E-03	
bmpr1ba	ENSDARG00000104100	1.79	4.72E-03	
smoc1	ENSDARG00000088255	4.76	1.45E-08	56
nr2f2	ENSDARG00000040926	-1.61	3.72E-07	
nedd9	ENSDARG00000089878	-1.29	4.20E-04	
pdgfaa	ENSDARG00000055505	1.74	5.91E-03	

Table S1. Genes identified as differentially expressed through the optic fissure closure process, in common across zebrafish and mouse data sets. DEGs at 32, 48 and 56 hpf in zebrafish were compared to DEGs identified in mouse over E10.5 to E12.5. Common genes are shown at each time point with associated Ensembl GeneID, the Log_2 fold change between optic fissure (OF) and dorsal retina (DR) tissue in zebrafish, and adjusted *p*-value (FDR).

Family	Patient	Gender	Ethnicity	Age	Gene	Ocular Phenotype	Systemic Features	BCVA , LogMAR	
								R	L
F1	1-3	M	Caucasian	17	*ANK3*	Bilateral iris and chorioretinal coloboma	Nil	2.20	0.44
F2	2-1	M	Black or Black British:Caribbean	5	*ANK3*	Bilateral optic nerve hypoplasia and left optic disc coloboma, left esotropia	Chiasmal hypoplasia with hypothalamo-pituitary abnormalities (septo-optic dysplasia	X	X
F3	3-2	F	Indian	40	*BMPR1B*	Left optic disc coloboma	Nil	X	X
	3-3	F	Indian	13	*BMPR1B*	Bilateral optic disc coloboma, nystagmus, small left esotropia	Nil	0.65	0.80
	3-4	F	Indian	18	*BMPR1B*	Bilateral optic disc coloboma, horizontal and pendular nystagmus, right moderate esotropia	Nil	1.50	0.20
F4	4-1	M	Caucasian	9	*BMPR1B*	Unilateral right microphthalmia with dense cataract and reported persistent hyperplastic primary vasculature (PHPV)	Nil	NPL	0.00
F5	5-3	M	Caucasian	10m	*BMPR1B*	Bilateral iris and chorioretinal coloboma	Nil	CSM	CSM
F6	6-3	M	Caucasian	4	*BMPR1B*	Bilateral iris, chorioretinal coloboma	Micrognathia, Autistic Behaviour, Attention Deficit Hyperactivity Disorder	0.00	1.00
F7	7-1	M	Caucasian	8	*PDGFRA*	Bilateral chorioretinal coloboma and microphthalmia	Global Developmental Delay, Autistic Behaviour, Delayed Gross Motor Development	-	-
F8	8-1	M	Caucasian	9	*CDH4*	Unilateral right iris coloboma	Intellectual Disability, Postnatal Microcephaly	-	-

Table S2. Demographics and clinical features of probands identified with novel potential pathogenic variants. Summary of each proband harbouring an SNV identified in the genes assessed. Best corrected visual acuity (BCVA) as Logarithm of the Minimum Angle of Resolution (LogMAR) was reported where available, with age/ethnicity/gender/ and clinical ocular phenotype reported. CSM, central steady and maintained vision; NPL, no perception to light.

Gene	cDNA / GRCh38	Protein	PM1	PM2	PP2	PP3
ANK3	NM_020987.5<br>c.11650 A>T<br>10:60069231:T:A	p.(Thr3884Ser)	-	< 0.002% in gnomAD exomes	-	+++
ANK3	NM_020987.5<br>c.3658 A>G<br>10:60086767:T:C	p.(Ile1220Val)	-	< 0.002% in gnomAD exomes	-	+++
BMPR1B	NM_001203.2<br>c.272 G>T<br>4:95115710:G:T	p.(Arg91Ile)	-	< 0.007% in gnomAD exomes	17/26 non-VUS missense variants pathogenic, 21/101 clinically reported variants pathogenic	+++
	NM_001203.2<br>c.1127 G>A<br>4:95148798:G:A	p.(Arg376His)	Protein domain has 15 non-VUS, pathogenicity 66.7%	Absent from controls		+++
	NM_001203.2<br>c.671 G>A<br>4:95129947:G:A	p.(Arg224His)		<0.096 in gnomAD exomes		++
	NM_001203.2<br>c.671 G>T<br>4:95129947:G:T	p.(Arg224Leu)		Absent from controls		+++
PDGFRA	NM_006206.6<br>c.1295 C>T<br>4:54272451:C:T	p.(Thr432Met)	-	< 0.002% in gnomAD exomes	530/1657 reported variants benign	+++
CDH4	NM_001794.5<br>c.1291 C>T<br>20:61910524:C:T	p.(Arg431Cys)	-	Absent from controls	13/16 clinically reported variants benign	++

Table S3. ACMG/AMP criteria met. Supporting information for classification of reported variants. PM1 is related to missense variants located in a hotspot region, PM2 relates to the presence in control populations including gnomAD exomes, 1000 genomes, PP2 missense variants in a gene has a low rate of being benign, and PP3 *in silico* predictions of pathogenicity.

5. Supplementary References

1. Rentzsch P, Witten D, Cooper GM, Shendure J, Kircher M. CADD: predicting the deleteriousness of variants throughout the human genome. Nucleic Acids Res. 2019;47(D1):D886-D894, doi:10.1093/nar/gky1016.
2. Tang K, Xie X, Park JI, Jamrich M, Tsai S, Tsai MJ. COUP-TFs regulate eye development by controlling factors essential for optic vesicle morphogenesis. Development. 2010;137(5):725-734, doi:10.1242/dev.040568.
3. Eberhart JK, He X, Swartz ME, et al. MicroRNA Mirn140 modulates Pdgf signaling during palatogenesis. Nat Genet. 2008;40(3):290-298, doi:10.1038/ng.82.
4. Matt N, Ghyselinck NB, Pellerin I, Dupe V. Impairing retinoic acid signalling in the neural crest cells is sufficient to alter entire eye morphogenesis. Dev Biol. 2008;320(1):140-148, doi:10.1016/j.ydbio.2008.04.039.
5. Gestri G, Bazin-Lopez N, Scholes C, Wilson SW. Cell Behaviors during Closure of the Choroid Fissure in the Developing Eye. Front Cell Neurosci. 2018;12:42, doi:10.3389/fncel.2018.00042.
